# Supplementary material for: Claustrophilic oviposition: Oviposition performance depends on container size in a novel forced oviposition method for Culex quinquefasciatus and Aedes aegypti
Source: PLoS Negl Trop Dis. 2025 Jul 14;19(7):e0013044. doi: 10.1371/journal.pntd.0013044 (PMC12279147; doi:10.1371/journal.pntd.0013044)
Supplement: Attachment — Submitted filename: 00 Response to Reviewers.docx [file pntd.0013044.s003.docx]

Response to comments from Reviewer(s):

After careful consideration, we feel that it has merit but does not fully meet PLOS Neglected Tropical Diseases's publication criteria as it currently stands. Therefore, we invite you to submit a revised version of the manuscript that addresses the points raised during the review process.

***We thank the reviewers for their positive assessment of the work and the helpful comments. Below are our responses for each comment/ suggestion. The reviewer comment is in normal Times New Roman 12pt font while our response is in **bold** following three stars (***). In the main text of the manuscript, we have made our changes visible with red font.

**Journal Requirements:**

1) Please ensure that the CRediT author contributions listed for every co-author are completed accurately and in full.
At this stage, the following Authors/Authors require contributions: Kendra A. Dagg, Alden S. Estep, Cason E. Bartz, and Edwin Burgess. Please ensure that the full contributions of each author are acknowledged in the "Add/Edit/Remove Authors" section of our submission form.
The list of CRediT author contributions may be found here: <https://journals.plos.org/plosntds/s/authorship#loc-author-contributions>

***** We have made the required change to the authors contributions list**

2) We do not publish any copyright or trademark symbols that usually accompany proprietary names, eg ©,  ®, or TM  (e.g. next to drug or reagent names). Therefore please remove all instances of trademark/copyright symbols throughout the text, including:
- ® on page: 7.

***** Page 7, Line 147 has been edited to reflect journal requirements**

3) Please upload all main figures as separate Figure files in .tif or .eps format. For more information about how to convert and format your figure files please see our guidelines: 
<https://journals.plos.org/plosntds/s/figures>

***** Figures formatted and submitted according to guidelines**

4) Some material included in your submission may be copyrighted. According to PLOSu2019s copyright policy, authors who use figures or other material (e.g., graphics, clipart, maps) from another author or copyright holder must demonstrate or obtain permission to publish this material under the Creative Commons Attribution 4.0 International (CC BY 4.0) License used by PLOS journals. Please closely review the details of PLOSu2019s copyright requirements here: PLOS Licenses and Copyright. If you need to request permissions from a copyright holder, you may use PLOS's Copyright Content Permission form.
Please respond directly to this email and provide any known details concerning your material's license terms and permissions required for reuse, even if you have not yet obtained copyright permissions or are unsure of your material's copyright compatibility. Once you have responded and addressed all other outstanding technical requirements, you may resubmit your manuscript within Editorial Manager. 
Potential Copyright Issues:

- Please confirm (a) that you are the photographer of Figures 1, 2, and 3, or (b) provide written permission from the photographer to publish the photo(s) under our CC BY 4.0 license.

***** Photographs were provided by the first author, Kendra A. Dagg. We have added a statement at the end of Figures 1-3 crediting the photographer.**

5) Please amend your detailed Financial Disclosure statement. This is published with the article. It must therefore be completed in full sentences and contain the exact wording you wish to be published. Please ensure that the funders and grant numbers match between the Financial Disclosure field and the Funding Information tab in your submission form. Note that the funders must be provided in the same order in both places as well.
- State the initials, alongside each funding source, of each author to receive each grant. For example: "This work was supported by the National Institutes of Health (####### to AM; ###### to CJ) and the National Science Foundation (###### to AM)."
- State what role the funders took in the study. If the funders had no role in your study, please state: "The funders had no role in study design, data collection and analysis, decision to publish, or preparation of the manuscript.".
If you did not receive any funding for this study, please simply state: u201cThe authors received no specific funding for this work.u201d

***** We have corrected the Financial Disclosure statement and author to receive each grant.**

**Reviewers' Comments:**

Reviewer's Responses to Questions

**Key Review Criteria Required for Acceptance?**

As you describe the new analyses required for acceptance, please consider the following:

**Methods:**

-Are the objectives of the study clearly articulated with a clear testable hypothesis stated?

-Is the study design appropriate to address the stated objectives?

-Is the population clearly described and appropriate for the hypothesis being tested?

-Is the sample size sufficient to ensure adequate power to address the hypothesis being tested?

-Were correct statistical analysis used to support conclusions?

-Are there concerns about ethical or regulatory requirements being met?

Reviewer #1: 1. The study’s aim of “comparing egg numbers” is not associated with a clearly defined threshold; it fails to distinguish expected outcomes for oviposition rate, egg number, and hatch rate.

***** Addressed by removing ‘…at each egg count’ in the sentence listing variables collected in Experiment 1 to clarify that egg numbers were not the primary aim of the study, and how any thresholds were defined. As a methods paper, the main objective of the study was to assess the usability of Morgan et al’s (2010) forced oviposition method for other mosquito vector species besides *Anopheles spp.* The authors have ensured that the listed data variables collected in the Methods are in order of precedence, with the first stating ‘did the female oviposit (yes/no)’.**

**Original text (Methods): Variables collected at each egg count included: 1) did the female oviposit (yes/no) (Fig 2A-B), 2) number of eggs laid, and 3) number of days between transfer to oviposition tube to laid eggs.**

**Revised text (Methods): Variables collected included: 1) did the female oviposit (yes/no) (Fig 2A-B), 2) number of eggs laid, and 3) number of days between transfer to oviposition tube to laid eggs.**

Additionally, potential confounding factors such as mosquito age or degree of blood engorgement were not controlled for in assessing oviposition performance.

***** We agree that these variables influence oviposition performance and have addressed this concern by adding a statement with reference Day 2016 in the Discussion. Due to difficulties in controlling these factors in field-caught mosquitoes, both variables were not monitored in the study. This was stated as a study limitation in the Discussion.**

**Original text (Discussion): It is worth noting that the longevity of eggs was not examined in this study.**

**Revised text (Discussion): It is worth noting that the age and degree of blood engorgement of laboratory and field-caught mosquitoes was not controlled for, nor was the longevity of eggs was not examined in this study. Adult age and level of blood engorgement are key factors that influence oviposition, however, it is difficult to control for these in field caught populations (Day 2016). Further study will need to be conducted to determine if age and blood engorgement significantly impact the success of this forced oviposition method.**

2. The definition and observation criteria for "claustrophilic" behavior lack rigorous quantification.

***** Addressed by adding a statement explaining how the authors define ‘claustrophilic behavior in oviposition’ in the Discussion. Here, the claustrophilic definition does not include quantifiable metrics and a defined ratio/criteria of space. We have defined the term by its etymology where “claustro” comes from the Latin word “claustrum” to mean “closed space” and “philic”, coming from the Greek word “philous” meaning affinity, attraction, or preference. This is important so as to not anthropomorphize the behavior. Additionally, in the view of the authors, we have chosen this term because it is both etymologically correct and enhances the attractiveness of the title to encourage readership and adoption of the method.**

**Original text: Both species demonstrated a claustrophilic behavior in oviposition, with more females laying in 1.5 and 5 mL tubes for *Cx. quinquefasciatus* and 1.5 mL tubes for *Ae. aegypti*.**

**Revised text: Both species appeared to demonstrate a claustrophilic behavior in oviposition (i.e. displaying an oviposition preference and higher frequency of ovipositing in smaller volume tubes by gravid females), with more females laying in 1.5 and 5 mL tubes for *Cx. quinquefasciatus* and 1.5 mL tubes for *Ae. aegypti*.**

3. The tube sizes used in Experiment 2 (1.5, 5, 15, and 50 mL) are not proportionally scaled or logically progressive, making it difficult to establish a consistent gradient for interpretation.

***** Concern addressed by adding a statement in the Discussion explaining criteria for equipment selection and how it presents as a study limitation.**

**Added text (Discussion):** One limitation in the present study for the claim that mosquitoes exhibit claustrophilic oviposition is that tubes were not selected based on a consistent ratio of increasing size. Instead, tube sizes were selected based on their similar material (i.e, plastic) and common availability in laboratories and mosquito control districts. Thus, with the data generated in the current study, we cannot rule out that tube dimensions may have played a role in oviposition behaviors. Further study, using containers proportionally scaled in size, would strengthen the definition of claustrophilic oviposition.

Furthermore, moisture conditions differed between species—Cx. tubes retained a surface water layer, whereas Ae. tubes had excess water removed—which could significantly affect oviposition behavior.

***** No changes made. We agree with the Reviewer that moisture levels in the tubes could significantly impact oviposition behavior. However, as a methods paper which aims to introduce an easy and quick method to readers of varying levels of insectary experience, we believe that adding restrictions and precise instructions on tube moisture levels would needlessly complicate the technique. Thus, methods and results reflect an outcome if this is not considered.**

The criteria for determining oviposition were not clearly described, including whether this was assessed visually and whether all germination papers were collected and systematically examined.

***** Addressed by adding statement explaining how oviposition was determined in the Methods.**

**Added text (Methods): Oviposition was determined through visual examination of germination papers and cotton under a dissecting microscope (Stemi 2000, Zeiss International, Germany).**

Reviewer #2: Yes the methods are well described and appropriate

***** We thank the reviewer for this positive feedback**

Reviewer #3: The nature of the wild mosquitos need more description (time collected, location, identification method...).

***** This information has been added, including a reference to identification keys by Darcie and Ward 2016 used to identify species. For operational and privacy purposes, the Gainesville Mosquito Control Department has requested that details on trap locations remain anonymous.**

**Added text: Mosquitoes were collected by the city Mosquito Control Department in July 2024 within the Gainesville, FL city area and were morphologically identified using keys from Darsie et al. [12].**

Was the tap water used dechlorinated, fluoridated, harvested rain water or borehole water?

***** Comment addressed by adding a brief descriptor of tap water supply. Exact parameters for water chemistry are unknown, but water is regularly used by this lab for rearing multiple mosquito strains, including those used in this study. To address the Reviewers concern regarding water quality, we added instruction outlining the importance of identifying appropriate water sources necessary for mosquito rearing.**

**Original text (Methods): Cotton balls were broken into smaller pieces and were moistened using tap water.**

**Revise text (Methods): Cotton balls were broken into smaller pieces and were moistened using Gainesville city tap water.**

**Additional text (Discussion): A key consideration for adopting this method is the quality and type of water that is available. While tap water was used in this study, if water quality is deemed unacceptable for rearing purposes, then alternative waters sources may be necessary.**

**Results**

-Does the analysis presented match the analysis plan?

-Are the results clearly and completely presented?

-Are the figures (Tables, Images) of sufficient quality for clarity?

Reviewer #1: (No Response)

Reviewer #2: Yes to the above

***** We thank the reviewer for this positive feedback**

Reviewer #3: Well presented and of sufficient quality.

***** We thank the reviewer for this positive feedback**

**Conclusions:**

-Are the conclusions supported by the data presented?

-Are the limitations of analysis clearly described?

-Do the authors discuss how these data can be helpful to advance our understanding of the topic under study?

-Is public health relevance addressed?

Reviewer #1: The conclusion that the mosquitoes “exhibited claustrophilic behavior” is not well-supported, as it is based solely on oviposition rates without behavioral recordings or exploratory data. The study also lacks discussion of potential effects of the method on mosquito longevity or offspring quality, and does not address possible impacts on sex ratio or population fitness in downstream research.

***** Reviewer brings up very good points, and we agree that further study is required to more accurately describe this particular oviposition behavior. Key limitations have been added into the Discussion along with a statement emphasizing the importance of further studying this behavior. This paper serves as the basis for introducing the forced oviposition method with instructions for labs to tailor this method to their own lab conditions. The addition of the suggested parameters are beyond the scope of this paper and would have resulted in a more complex project and longer manuscript that would shift focus away from the main objective. While mosquito longevity and offspring quality were not monitored in this study, statements were added in the Methods and Discussion on how long hatched larvae were reared prior to disposal.**

**Added text (Methods):** **Any hatched larvae were reared up to the L2 stage for ease of visualization.**

**Added text (Discussion): All larvae that hatched were reared to the L2 stage at a growth rate commonly observed in both laboratory and field-caught strains of these species [19,20]**

Reviewer #2: yes to the above

***** We thank the reviewer for this positive feedback**

Reviewer #3: (No Response)

**Editorial and Data Presentation Modifications?**

Use this section for editorial suggestions as well as relatively minor modifications of existing data that would enhance clarity. If the only modifications needed are minor and/or editorial, you may wish to recommend “Minor Revision” or “Accept”.

Reviewer #1: (No Response)

Reviewer #2: (No Response)

Reviewer #3: (No Response)

**Summary and General Comments:**

Use this section to provide overall comments, discuss strengths/weaknesses of the study, novelty, significance, general execution and scholarship. You may also include additional comments for the author, including concerns about dual publication, research ethics, or publication ethics. If requesting major revision, please articulate the new experiments that are needed.

Reviewer #1: This study proposed and evaluated a modified forced oviposition method applicable to Culex quinquefasciatus and Aedes aegypti, finding that both species exhibited higher oviposition success rates in small-volume containers and displayed "claustrophilic" oviposition behavior. The method uses inexpensive materials and a single-mosquito setup, making it suitable for oviposition induction in both laboratory and wild mosquito populations, and holds promise for facilitating colony establishment and genetic experiments in vector research.

The background lacks a systematic review of existing forced oviposition methods, their limitations, and comparative analyses. The "claustrophilic" hypothesis was introduced late in the manuscript instead of being explicitly stated and biologically contextualized in the introduction. There is no literature-based or physiological support for the mechanism of “small space–induced oviposition,” making the hypothesis largely speculative.

***** Comments addressed by adding citations in the Introduction of forced-oviposition used successfully and a statement regarding the lack of information on any forced-oviposition methods available for mosquito vectors besides *Anopheles spp.* Limitations on similar methods found in the literature are briefly explained in the Discussion. As there is very little literature available introducing similar methods being used for *Aedes spp.* and *Culex spp.*, it was determined there was not enough to conduct any comparative analyses of techniques. Edited the objectives to ensure they are more explicit and introduce the ‘claustrophilic’ hypothesis in the Introduction.**

**Added text (Introduction): The Morgan et al. [10] method has been successfully used to establish wild and laboratory colonies for multiple *Anopheles* spp. in Thailand, Ethiopia, Madagascar, and Uganda [9–12]. However, there is no literature on similar techniques available used to establish colonies of other mosquito vector species.**

**Original text (Introduction): Additionally, Morgan et al. [10] suggested that the success of the method could be related to the confined space the mosquitoes are put in.**

**Revised text (Introduction): Additionally, Morgan et al. [10] hypothesized that the success of the method could be related to the confined space the mosquitoes are put in which may suggest an affinity for tight spaces (i.e. claustrophilic) when ovipositing.**

Reviewer #2: This is a very well written detailed paper that can be useful for programs that has important implications for enahcing vector-borne disease research. Minor edits are:

Line 108 and 109 in Materials and Methods: Culex and Aedes already mentioned so can abbreviate the genus

***** Change incorporated**

**Original text:** **Laboratory reared strains and locally collected wild populations of *Culex quinquefasciatus* and *Aedes aegypti* were used in this study.**

**Revised text: Laboratory reared strains and locally collected wild populations of *Cx. quinquefasciatus* and *Ae. aegypti* were used in this study.**

Line 290 aegypti is capitalized

***** Change incorporated**

**Original text: In both *Cx. quinquefasciatus* and *Ae. Aegypti*, …**

**Revised text: In both *Cx. quinquefasciatus* and *Ae. aegypti,* …**

Could explain in line 307 what claustrophilic behavior is

***** Change incorporated. Added in a statement explaining how the authors define ‘claustrophilic behavior in oviposition’ in Discussion.**

**Original text: Both species demonstrated a claustrophilic behavior in oviposition, with more females laying in 1.5 and 5 mL tubes for *Cx. quinquefasciatus* and 1.5 mL tubes for *Ae. aegypti*.**

**Revised text: Both species appeared to demonstrate a claustrophilic behavior in oviposition (i.e. displaying an oviposition preference and higher frequency of ovipositing in smaller volume tubes by gravid females), with more females laying in 1.5 and 5 mL tubes for *Cx. quinquefasciatus* and 1.5 mL tubes for *Ae. aegypti*.**

Reviewer #3: The study presents a practical forced oviposition method adapted for Culex and Aedes species adapted from Anopheles methods highlighting its usefulness in generating sufficient F1 eggs for colony establishment despite their differing natural oviposition behaviors.

It is well written and easy for the reader to follow through and replicate the experiments.
The technique’s simplicity—requiring minimal female mosquitoes and low-cost lab materials—makes it highly accessible for vector control programs and various research settings.

A more elaborate description of the wild strains used will enhance repeatability and reveal any confounding factors on the outcome of the experiments including habitat type and environmental conditions pre-testing that might affect behavioral traits.

***** Change incorporated with compromise (refer to above response to Reviewer 3 under Methods section). We have added information on time of year and city, state field mosquitoes were trapped. Additionally, we have included a reference used for morphological identification. For operational and privacy purposes, the Gainesville Mosquito Control Department has requested that details on trap locations remain anonymous.**

**Added text: Mosquitoes were collected by the city Mosquito Control Department in July 2024 within the Gainesville, FL city area and were morphologically identified using keys from Darsie et al. [12].**

**Additional Comments:**

Additional Reviewer comments added in the body of the PDF file titled “PNTD-D-25-00520_reviewer” are addressed below:

Line 114: Expound on location, time of collection and confirmation of identity of the collected specimen.

***** Change incorporated. Refer to Methods section Reviewer 3 comment and author’s response.**

Line 120-121: Revise title headings to omit ‘Experiment 1, 2…’

***** We did not delete the Experiment 1 and 2 indicators in the Methods and Results sub-headings because it provides a method of quick and accurate reference for the authors when describing results and outlining the discussion. Additionally, we believe this ensures the paper is easy to follow and assists in guiding the reader between the two sets of data and multiple discussion points. We feel this is important because, although Experiment 1 and 2 are different, they use a lot of overlapping methodology.**

Line 139: Change ‘hr’ to ‘hrs’

***** Change incorporated**
